# A Simulation Analysis of an Extension of One-Dimensional Speckle Correlation Method for Detection of General In-Plane Translation

**DOI:** 10.1155/2014/704368

**Published:** 2014-01-30

**Authors:** Ivana Hamarová, Petr Šmíd, Pavel Horváth, Miroslav Hrabovský

**Affiliations:** ^1^Joint Laboratory of Optics of Palacky University and Institute of Physics of the Academy of Sciences of the Czech Republic, Institute of Physics of the Academy of Sciences of the Czech Republic, 17. listopadu, 50a, 77207 Olomouc, Czech Republic; ^2^Regional Centre of Advanced Technologies and Materials, Joint Laboratory of Optics of Palacky University and Institute of Physics of the Academy of Sciences of the Czech Republic, Faculty of Science, Palacky University, 17. listopadu, 12, 77146 Olomouc, Czech Republic

## Abstract

The purpose of the study is to show a proposal of an extension of a one-dimensional speckle correlation method, which is primarily intended for determination of one-dimensional object's translation, for detection of general in-plane object's translation. In that view, a numerical simulation of a displacement of the speckle field as a consequence of general in-plane object's translation is presented. The translation components *a*_*x*_ and *a*_*y*_ representing the projections of a vector **a** of the object's displacement onto both *x*- and *y*-axes in the object plane (*x*, *y*) are evaluated separately by means of the extended one-dimensional speckle correlation method. Moreover, one can perform a distinct optimization of the method by reduction of intensity values representing detected speckle patterns. The theoretical relations between the translation components *a*_*x*_ and *a*_*y*_ of the object and the displacement of the speckle pattern for selected geometrical arrangement are mentioned and used for the testifying of the proposed method's rightness.

## 1. Introduction

Speckle phenomenon [1], which became important especially after expansion of lasers, has found many applications in different scientific fields such as medicine (e.g., examination of eye refraction [2]), biology (e.g., measurement of biological activity [3]), and astronomy (e.g., measurement of diameter of astronomical objects [4]). Another application can be found in mechanics in measurement of the roughness [5], shape [6], and slope [7] of the object under investigation. Speckle effect also plays a significant role in continuum mechanics especially in measuring of small changes of a state of an object's surface [6–11]. The deformation state of an elementary area of the object's surface can be determined by the small deformation tensor with translation, rotation, and deformation components (*a*_*x*_, *a*_*y*_, *a*_*z*_), (*Ω*_*x*_, *Ω*_*y*_, *Ω*_*z*_), and (*ε*_*xx*_, *ε*_*xy*_, *ε*_*yy*_), respectively [9].

The paper deals particularly with determination of the object's displacement in the *x*-axis direction **a** = (*a*_*x*_, 0) as well as in both *x*-axis and *y*-axis direction **a** = (*a*_*x*_, *a*_*y*_), respectively. The other components of small deformation tensors are neglected; that is, the object is moving along the appropriate coordinate axes *x* and *y* as a rigid body. For determination of the object's displacement, we use the speckle correlation method proposed by Yamaguchi [12]. In this method, the cross-correlation function *R*_12_ of two intensities *I*_1_ and *I*_2_ representing speckle patterns detected before and after object translation is computed. A vector **A** = (*A*_*x*′_, *A*_*y*′_) gives a position of the maximum of the cross-correlation function *R*_12_ of the intensities *I*_1_ and *I*_2_, which corresponds to recorded displacement of the speckle pattern in a scan area of the detector. Stated vector **A** of the speckle pattern displacement is used to evaluate the object's translation vector **a** regressively [11].

The speckle correlation method is verified in one direction experimentally [10, 13] as well as through computer simulation [14], while the speckle pattern is captured in one row of a detector. The position *A*_*x*′_ of the maximum of the one-dimensional cross-correlation function of intensity signals of the speckle pattern responds to speckle pattern movement. The other translation components are not evaluated, because only one direction of object's surface motion along *x*-axis is assumed.

Nevertheless, it is possible to evaluate two translation components *a*_*x*_ and *a*_*y*_ from two speckle pattern movement components *A*_*x*^′^_ and *A*_*y*^′^_ acquired simultaneously by two-dimensional cross-correlation function [15], although as the paper shows, both components *A*_*x*^′^_ and *A*_*y*^′^_ can be also detected individually by means of one-dimensional cross-correlation function of intensities recorded from one row vector and one column vector oriented to appropriate axis direction. These vectors are results of a two-dimensional signal processing, which is introduced in detail in the paper.

An integral part of investigation of feasibility of speckle metrology is a numerical simulation. It enables studying the problems under different conditions, before being investigated in the laboratory. A numerical simulation of origin and propagation of speckle field is traditionally realized by computation of the Fresnel-Kirchhoff diffraction integral [16–19], which is transformed in such a way that Fast Fourier Transform (FFT) implementation is enabled [17–20]. Although FFT involves fast numerical evaluation of the diffraction integral, the main disadvantage is that sampling of the output plane is influenced by number of samples *m* × *n* in input plane. Further, this solving of diffraction problem is restricted only to special case, when an observation plane is parallel to the diffracting plane. For these reasons, the Fresnel-Kirchhoff diffraction integral without FFT application is computed in this paper.

The basic concept of the numerical simulation of propagation of the speckle field as well as modification of the model for pure in-plane object's translation is proposed in papers [14, 21]. The speckle field is generated by illumination of the object's surface, which is represented by a submatrix of *m*_*i*_ × *n*_*i*_ elements. The in-plane object's translation is simulated by shifting the submatrix by a number of columns. Corresponding speckle pattern displacement is evaluated through speckle correlation method. The object plane is parallel to the detection plane in [14]. More common situation in which the detection plane is rotated by the nonzero angle toward the object plane is considered in [21]. The above mentioned papers are concentrated on object's translation along *x*-axis direction being represented by only one translation component *a*_*x*_; thus **a** = (*a*_*x*_, 0).

In this paper, the object is translated along the *x*-axis, **a** = (*a*_*x*_, 0), as well as along both *x*- and *y*-axes, **a** = (*a*_*x*_, *a*_*y*_). The object's translation in the range from 10 **μ**m to 100 **μ**m is simulated by the proposed numerical model with selected input parameters.

The aim of the paper is to present an acceptable way of extension of the one-dimensional speckle correlation method primarily used for detection of one-dimensional object's translation to evaluate both translation components *a*_*x*_ and *a*_*y*_. Moreover, as is shown, the proposed method for determination of the translation components based on the one-dimensional cross-correlation function enables distinct optimization of the method by reduction of intensity values representing detected speckle patterns. Hence, the main advantage of the presented method rests on possibility of evaluation of the translation components from smaller amount of detected intensity values. Numerical results of determination of the translation components *a*_*x*_ and *a*_*y*_ of the object under test are compared with results obtained from theoretical relations.

## 2. Theory Description

**Figure 1:**
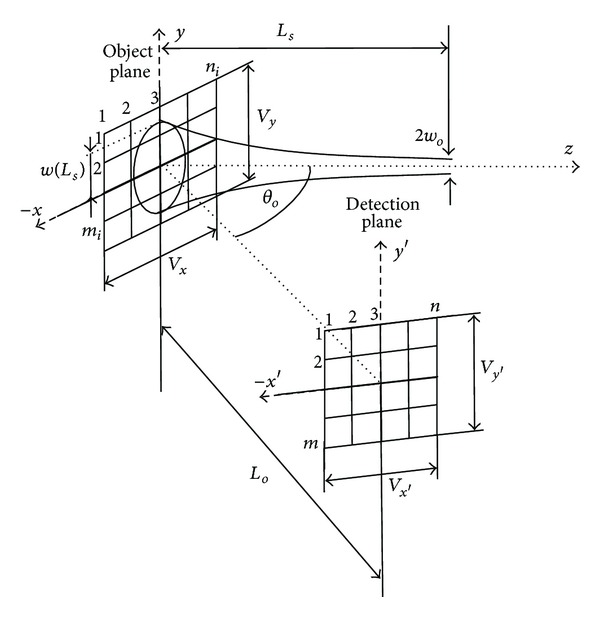
Geometrical arrangement for speckle pattern observation. Gaussian beam is incident on the object's surface of size *V*_*x*_ × *V*_*y*_ sampled by *m*_*i*_ × *n*_*i*_ points. The radii of the Gaussian beam at its waist and at the distance *L*_*s*_ from the waist are *ω*_*o*_ and *ω*(*L*_*s*_), respectively. Reflected field is detected by the matrix detector of size *V*_*x*′_ × *V*_*y*′_ and a number of points *m* × *n* at the distance *L*_*o*_ and the observation angle *θ*_*o*_.

Let us consider that an object of size *V*_*x*_ × *V*_*y*_ placed in the plane (*x*, *y*) is illuminated by a Gaussian beam as is depicted in Figure 1. The illuminated area of the object's surface is sampled by *m*_*i*_ × *n*_*i*_ points. The complex amplitude of the Gaussian beam in the object's surface with random variable surface roughness Δ(*x*, *y*) can be determined from the following relation [16, 21]:

(1) U(x,y,Δ(x,y)) =ωoω(Ls+Δ(x,y))exp⁡[−x2+y2ω2(Ls+Δ(x,y))]  ×exp⁡[−ik(Ls+Δ(x,y))   −ikx2+y22R(Ls+Δ(x,y))+iξ(Ls+Δ(x,y))],

where *R* is a radius of curvature of the wavefront comprising the beam, *ξ* is a longitudinal phase delay of the beam, and *ω*_*o*_ and *ω* are radii of the Gaussian beam at its waist and at a distance *L*_*s*_ from the waist (Figure 1).

To simulate reflection from the object's surface, the complex amplitude *U*(*x*, *y*) of the Gaussian beam is multiplied by the phase factor exp⁡[−*ik*(Δ(*x*, *y*))], where *k*Δ(*x*, *y*) is a random phase shift caused by surface roughness Δ(*x*, *y*). The resulting complex amplitude *U*(*x*′, *y*′) in the detection plane (*x*′, *y*′) at the distance *L*_*o*_ from the object plane (*x*, *y*) is determined by the following relation [16, 21]:

(2) U(x′,y′)=exp⁡(−ikLoN)iλLoN×∫−∞∞∫−∞∞U(x,y)exp⁡(−ikΔ(x,y))   ×(−ik(xN−x′)2+(y−y′)22LoN)dxdy,

where

(3) $$\begin{array}{c} x_{N}=x\cos\theta_{o},\\ L_{oN}=L_{o}-x_{N}\tan\theta_{o} \end{array}$$

represent transform relations of the *x*-coordinate in the object plane (*x*, *y*) and the distance *L*_*o*_ for the case of the object plane is rotated around the *y*-axis by the angle *θ*_*o*_ against the *x*-axis (Figure 1) [21]. The matrix detector of size *V*_*x*′_ × *V*_*y*′_ placed in the detection plane (*x*′, *y*′) is sampled by *m* × *n* points.

Now, let us consider a rigid body moving along the *x*-axis; thus the vector **a** = (*a*_*x*_, 0) of the object's translation has only one nonzero component *a*_*x*_. Then the following relation between translation component *a*_*x*_ and the *x*-component *A*_*x*′_ of the speckle pattern displacement vector **A** = (*A*_*x*′_, 0) can be derived [10]:

(4) $$\begin{array}{c} A_{x^{\prime }}=a_{x}\left(\frac{L_{o}}{L_{s}\cos\theta_{o}}+\cos\theta \right), \end{array}$$

whereas the *y*-component *A*_*y*′_ = 0.

Further, if the object is subject to general in-plane motion described by the vector **a** = (*a*_*x*_, *a*_*y*_), then *a*_*y*_ ≠ 0 and the component *A*_*y*′_ of the speckle pattern displacement vector **A** = (*A*_*x*′_, *A*_*y*′_) can be derived [10] as

(5) $$\begin{array}{c} A_{y^{\prime }}=a_{y}\left(\frac{L_{o}}{L_{s}}+1\right). \end{array}$$

Both components *A*_*x*′_ and *A*_*y*′_ determine the position of maximum of the two-dimensional cross-correlation function of intensity sets (speckle patterns) detected before and after object's translation *a* = (*a*_*x*_, *a*_*y*_) [15]. The *x*- and *y*-translation components *a*_*x*_ and *a*_*y*_ can be computed from (4) and (5) after substituting the acquired components *A*_*x*′_ and *A*_*y*′_, as well as geometrical parameters *L*_*o*_, *L*_*s*_, and *θ*_*o*_ of the optical arrangement (Figure 1).

**Figure 2:**
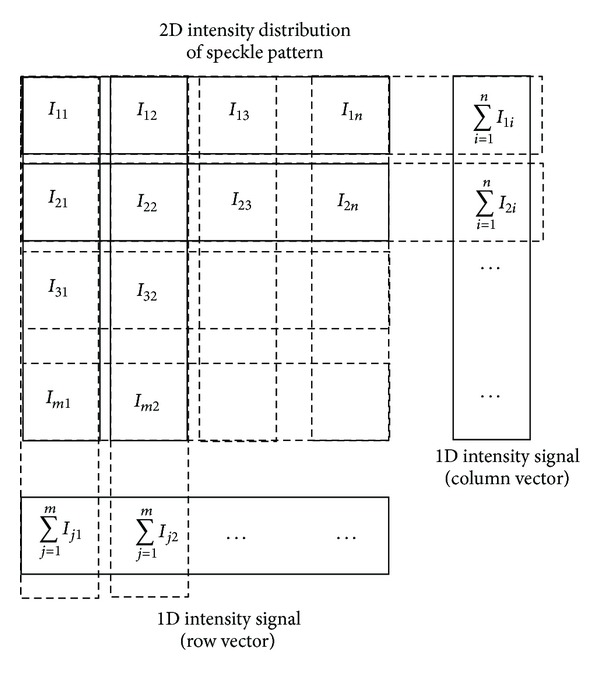
The principle of numerical processing of 2D speckle pattern to acquire 1D intensity signal for successive 1D numerical cross-correlation.

Nevertheless, in this paper, a possibility to evaluate each translation components *a*_*x*_ and *a*_*y*_ separately by the one-dimensional (1D) cross-correlation function of intensity signals of speckle patterns is mentioned, although, as numerical simulation shows, this approach exploiting the 1D cross-correlation requires preliminary numerical processing of the detected two-dimensional (2D) speckle patterns at first (see Figure 2). This numerical processing includes sums of intensities within each column of a detector returning a row intensity vector in case of *a*_*x*_ component evaluation and sums of intensities within each row of the detector returning a column intensity vector in the case of *a*_*y*_ component evaluation, respectively. Then the original 2D intensity signal is reduced to two 1D intensity signals (row and column). Corresponding signals obtained from subsequent 2D speckle patterns can be processed through the 1D cross-correlation.

The following text deals with achieved numerical results of simulation of translation of the object and successive evaluation of the translation by the 1D cross-correlation of intensity signals. Firstly, the numerical preprocessing of 2D intensity distribution of speckle pattern is not used. The row and column intensity signals are represented by arbitrarily selected same row and column, respectively, of the 2D intensity distribution of speckle pattern detected before and after object's translation (Section 3.1). The results of Section 3.1 show that one row and column do not suffice for correct determination of general object's translation. Therefore, the other way of the evaluation of the object's translation is applied. The row and column intensity signals are acquired by the above described way of the numerical signal processing of 2D intensity distribution of speckle pattern (Section 3.2). Subsequently, decimation [22] of the 2D intensity signal, which is based on reduction of resolution of the 2D intensity signal by skipping of certain amount of intensity values within rows and columns of the detector, is used. The signal processing described in Section 3.2 is then applied on the decimated (reduced) 2D intensity signal (Section 3.3).

## 3. Achieved Numerical Results and Discussion

### 3.1. Evaluation of the Translation Components *a*_*x*_ and *a*_*y*_ from One Selected Row and Column of the Detector

The achieved numerical results of evaluation of object's translation components *a*_*x*_ and *a*_*y*_ by means of 1D cross-correlation function of intensities of arbitrary selected row and column are summarized in Tables 1 and 2. To make a comparison, two speckle patterns with different mean speckle sizes *α*_*x*′_, *α*_*y*′_ oriented in both *x*- and *y*-axes directions are investigated. The speckle sizes *α*_*x*′_, *α*_*y*′_, which are within numerical procedure regulated by change in the radius *ω*_*o*_ at the waist of a Gaussian beam, are computed from autocorrelation function of intensity according to [23].  Table 1 stands for mean speckle sizes *α*_*x*′_ = 264.9 **μ**m and *α*_*x*′_ = 225.5 **μ**m (*ω*_*o*_ = 60 **μ**m) and Table 2 stands for mean speckle sizes *α*_*x*′_ = 133.6 **μ**m and *α*_*y*′_ = 117.7 **μ**m (*ω*_*o*_ = 30 **μ**m).

The first two columns of the tables contain the object's translation components *a*_*x*_, *a*_*y*_ within the ranges *a*_*x*_∈[10, 100] **μ**m, *a*_*y*_ = 0 **μ**m (upper part of the tables) and *a*_*x*_∈[10, 100] **μ**m, *a*_*y*_∈[10, 100] **μ**m (lower part of the tables). Stated (detected) speckle pattern displacements *A*_*x*′_ and *A*_*y*′_ (the fifth and sixth column) are compared with displacements *A*_*x*′_^theor^and *A*_*y*′_^theor^ of speckle pattern computed theoretically by means of (4) and (5). The seventh and eighth columns contain the maximum of the normalized cross-correlation function *r*_12_ of intensity signals *I*_1_ and *I*_2_ captured in individual row and column of the detector. Finally, the last two columns contain object's translation components *a*_*x*_ and *a*_*y*_ computed by means of (4) and (5) after substituting the detected values *A*_*x*′_ and *A*_*y*′_. For the sake of clarity, the values in columns 1 and 2, as well as the corresponding values in columns 9 and 10, are highlighted by bold font.

Initial parameters of the computer simulation are as follows: the wavelength of light *λ* = 632.8 nm, the size of an illuminated area of the object's surface *V*_*x*_ = *V*_*y*_ = 4 mm sampled by *m*_*i*_ × *n*_*i*_ = 400 × 400 points, the size of a row (column) of the matrix detector *V*_*x*′_ = 8 mm, and *V*_*y*′_ = 8 mm sampled by *m*(*n*) = 570 points. The distance *L*_*o*_ between the object plane (*x*, *y*) and the detection plane (*x*′, *y*′) is *L*_*o*_ = 0.4 m, the distance *L*_*s*_ between the waist of the Gaussian beam and the object plane (*x*, *y*) is *L*_*s*_ = 0.2 m, the angle of observation is *θ*_*o*_ = 30° and the radius of the Gaussian beam at its waist is *ω*_*o*_ = 60 **μ**m (Table 1), and *ω*_*o*_ = 30 **μ**m (Table 2).

Let us firstly discuss results of evaluation of object's translation components *a*_*x*_ and *a*_*y*_ in the ranges *a*_*x*_∈[10, 100] **μ**m and *a*_*y*_ = 0 **μ**m (upper part of Tables 1 and 2). Such translation components correspond to the case when the object under test translates in the *x*-axis direction only. Good results are achieved only for evaluation of *a*_*x*_ component as can be seen by comparing column one and column nine. In addition, the appropriate maximum *r*_12_(*A*_*x*′_) of cross-correlation function remains relatively high as speckle pattern displacement *A*_*x*′_ increases. On the other hand, as regards the evaluation of *a*_*y*_ component, the values from the last column randomly fluctuate with the displacement *A*_*x*′_ and maximum *r*_12_(*A*_*y*′_) of cross-correlation function firstly decreases as displacement *A*_*x*′_ increases and then begins to fluctuate as well.

**Figure 3:**
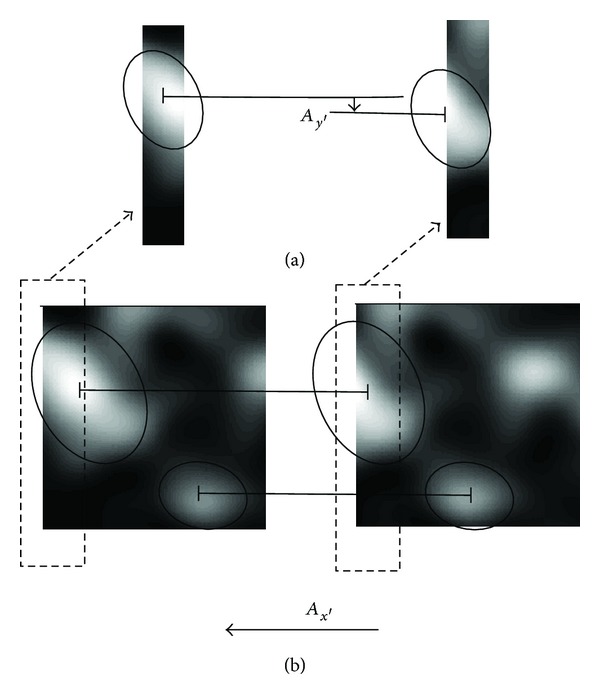
Displacement of speckle pattern detected (a) in a selected narrow rectangular area of a detector, (b) in the whole area of the detector. Black ovals delimit shape of individual speckles. Speckle pattern is only shifted by *A*_*x*′_, whereas *A*_*y*′_ = 0. However, the speckle pattern displacement *A*_*y*′_ evaluated in the case (a) is *A*_*y*′_ ≠ 0.

Nevertheless, one could predict this behavior, because displacement *A*_*x*′_ of speckle pattern along the *x*′-axis direction causes simultaneous change in speckle patterns recorded in the detector oriented perpendicularly to the direction of the speckle displacement *A*_*x*′_ before and after the displacement. This change can be evaluated as a spurious speckle pattern displacement along the *y*′-axis direction in one column or, more generally, in selected narrow rectangular area of the detector, as is depicted in the Figure 3.

Further, let us go through the results of evaluation of *a*_*x*_ and *a*_*y*_ components, both in the range [10, 100] **μ**m (lower part of Tables 1 and 2). Both evaluated translation values from column nine and column ten of the tables differ from the assumed values of *a*_*x*_ and *a*_*y*_ and begin to fluctuate as speckle displacements *A*_*x*′_ and *A*_*y*′_ increase. The maxima *r*_12_(*A*_*x*′_) and *r*_12_(*A*_*y*′_) of cross-correlation function firstly decrease as speckle displacements *A*_*x*′_ and *A*_*y*′_ increase and then begin to fluctuate as well. For the explanation of such strange behavior, the principle depicted in Figure 3 can be generalized for this case of 2D object's translation as well.

From the obtained results of numerical simulation, it is obvious that one row and one column of intensities with detected speckle pattern are not sufficient for detection of individual translation components of an object subject to general in-plane translation. In Section 3.2 the way of signal processing of a 2D speckle pattern into acquired 1D signal for successive one-dimensional numerical cross-correlation of intensities described in the Section 2 is applied.

### 3.2. Evaluation of the Translation Components *a*_*x*_ and *a*_*y*_ Exploiting the 2D Signal Processing before the 1D Numerical Correlation

**Figure 4:**
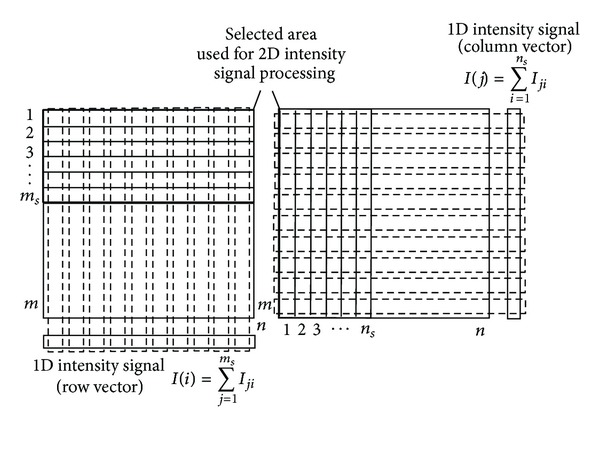
Illustration of 2D intensity signal processing for selected area of a detector.

Let us assume that the object is subject to general in-plane motion described by the vector **a** = (*a*_*x*_, *a*_*y*_) as well as pure *x*-axis motion **a** = (*a*_*x*_, 0). Selected translation components are in sequence *a*_*x*_ = *a*_*y*_ = 10 *μ*m and *a*_*x*_ = *a*_*y*_ = 100 **μ**m in the case of motion described by the vector **a** = (*a*_*x*_, *a*_*y*_) and *a*_*x*_ = 10 **μ**m, *a*_*x*_ = 100 **μ**m, and always *a*_*y*_ = 0 **μ**m in the case of motion described by the vector **a** = (*a*_*x*_, 0). Further, let us particularly aim at evaluation of the components *a*_*x*_ and *a*_*y*_ for the case of *a*_*x*_ = *a*_*y*_ ≠ 0 and the component *a*_*y*_ for the case of *a*_*x*_ ≠ 0 and *a*_*y*_ = 0. In contrast to Section 3.1, the speckle pattern is not detected only by one single row (column) of the detector but is rather detected by the whole matrix of the detector of size *m* = *n* = 570. For evaluation of the component *a*_*x*_, intensity values in each column of the detector are summed while a row intensity vector is returned and for evaluation of the component *a*_*y*_, intensity values in each row of the detector are summed while a column intensity vector is returned (Figure 2). Summing intensities over all elements of the columns or rows is not necessary but only part of the matrix detector is enough for the 2D intensity signal processing.  Figure 4 illustrates selected submatrix of size *m*_*s*_ × *n* (left) or *m* × *n*_*s*_ (right) used for the 2D intensity signal processing.

**Figure 5:**
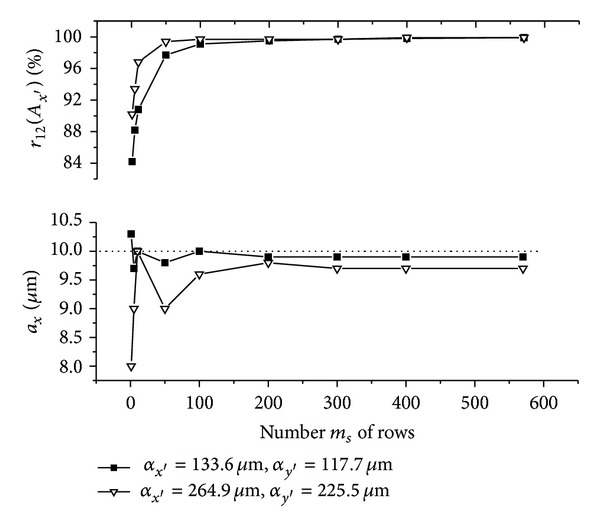
Resultant values of the evaluated object's translation component *a*_*x*_ as well as the maximum *r*_12_(*A*_*x*′_) of the 1D normalized cross-correlation function *r*_12_ of the intensity signals *I*_1_ and *I*_2_ as a function of number *m*_*s*_ of rows of the area used for the 2D intensity signal processing. The object is translated by *a*_*x*_ = *a*_*y*_ = 10 **μ**m.

**Figure 6:**
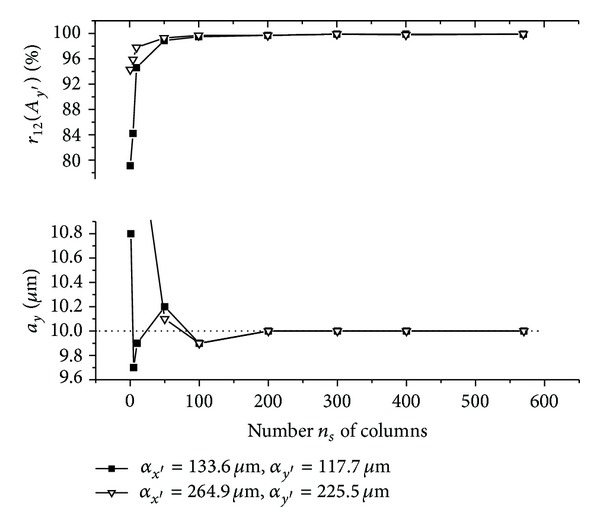
Resultant values of the evaluated object's translation component *a*_*y*_ as well as the maximum *r*_12_(*A*_*y*′_) of the 1D normalized cross-correlation function *r*_12_ of the intensity signals *I*_1_ and *I*_2_ as a function of number *n*_*s*_ of rows of the area used for the 2D intensity signal processing. The object is translated by *a*_*x*_ = *a*_*y*_ = 10 **μ**m.

**Figure 7:**
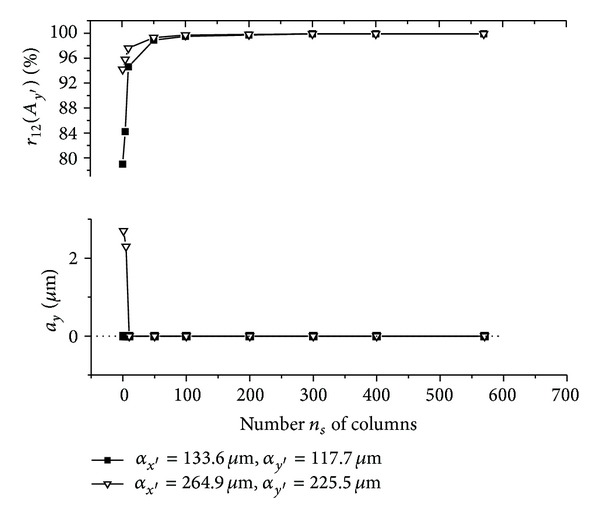
Resultant values of the evaluated object's translation component *a*_*y*_ as well as the maximum *r*_12_(*A*_*y*′_) of the 1D normalized cross-correlation function *r*_12_ of the intensity signals *I*_1_ and *I*_2_ as a function of number *n*_*s*_ of rows of the area used for the 2D intensity signal processing. The object is translated by *a*_*x*_ = 10 **μ**m and *a*_*y*_ = 0 **μ**m.

**Figure 8:**
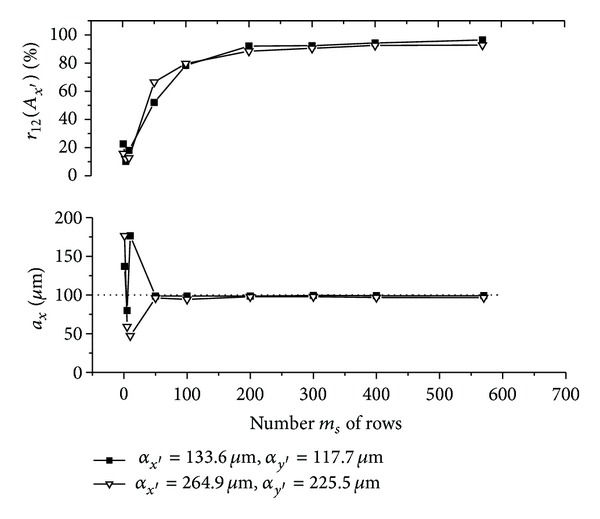
Resultant values of the evaluated object's translation component *a*_*x*_ as well as the maximum *r*_12_(*A*_*x*′_) of the 1D normalized cross-correlation function *r*_12_ of the intensity signals *I*_1_ and *I*_2_ as a function of number *m*_*s*_ of rows of the area used for the 2D intensity signal processing. The object is translated by *a*_*x*_ = *a*_*y*_ = 100 **μ**m.

**Figure 9:**
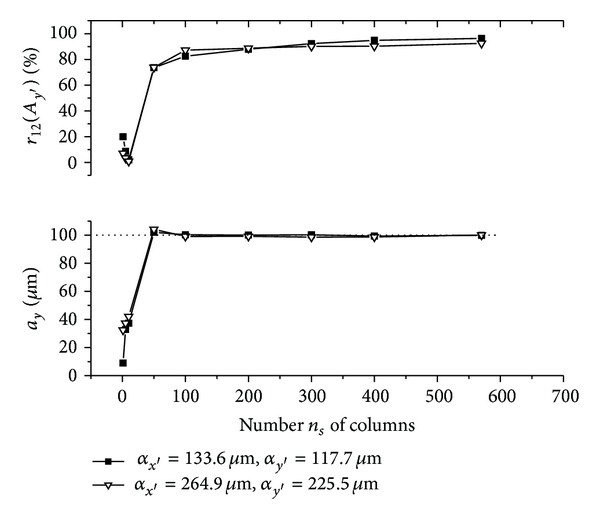
Resultant values of the evaluated object's translation component *a*_*y*_ as well as the maximum *r*_12_(*A*_*y*′_) of the 1D normalized cross-correlation function *r*_12_ of the intensity signals *I*_1_ and *I*_2_ as a function of number *n*_*s*_ of rows of the area used for the 2D intensity signal processing. The object is translated by *a*_*x*_ = *a*_*y*_ = 100 **μ**m.

**Figure 10:**
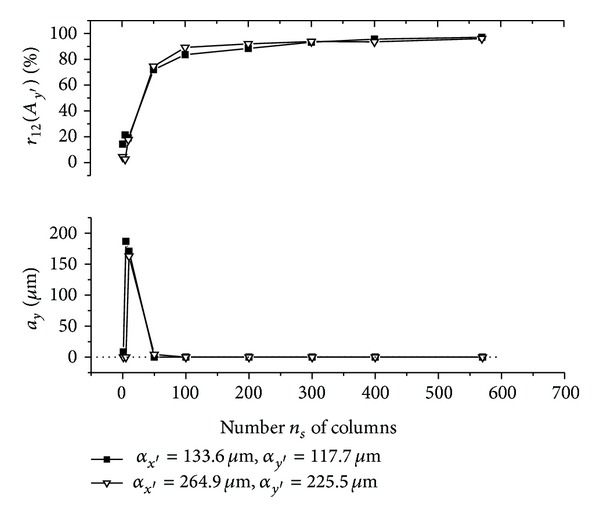
Resultant values of the evaluated object's translation component *a*_*y*_ as well as the maximum *r*_12_(*A*_*y*′_) of the 1D normalized cross-correlation function *r*_12_ of the intensity signals *I*_1_ and *I*_2_ as a function of number *n*_*s*_ of rows of the area used for the 2D intensity signal processing. The object is translated by *a*_*x*_ = 100 **μ**m and *a*_*y*_ = 0 **μ**m.

The graphs (Figures 5–10) show behavior of both evaluated object's translation components *a*_*x*_ and *a*_*y*_ and the maximum of the 1D normalized cross-correlation function *r*_12_ of intensity signals *I*_1_ and *I*_2_ as a function of number *m*_*s*_ (*n*_*s*_) of rows (columns) of the processed submatrix. The 1D signals *I*_1_ and *I*_2_ are obtained from 2D intensity signal processing of speckle pattern detected before and after object's translation. The black square marks stand for smaller speckle sizes (*α*_*x*′_ = 133.6 **μ**m and *α*_*y*′_ = 117.7 **μ**m) and white triangle marks stand for larger speckle sizes (*α*_*x*′_ = 264.9 **μ**m and *α*_*y*′_ = 225.5 **μ**m).

As can be seen, good results are achieved for *m*_*s*_, *n*_*s*_ ≥ 100. All evaluated translation components are approximately equal to the assumed ones. In addition, maxima of normalized cross-correlation function *r*_12_ are sufficiently high, although the use of speckle patterns with smaller speckle sizes makes the result more accurate. The reason is that, in general, a large number of speckles in speckle pattern are necessary for meaningful statistical evaluation [24]. The smaller speckle sizes fit the condition much better, because the area of the detector captures larger number of speckles.

As could be also noted, reasonable results are achieved also for *m*_*s*_, *n*_*s*_ = 50. For *m*_*s*_, *n*_*s*_ < 50, the evaluated object's translation components as well as stated maxima of cross-correlation function fluctuate with *m*_*s*_, *n*_*s*_. This behavior can be described as follows.

Let us suppose that object is translated purely along *x*-axis direction and only the translation component *a*_*y*_ is evaluated (see Figures 7 and 10). After the object's translation, some new values of intensity of speckle pattern occur on one side of detected area, whereas some values of intensity vanish on the other side of detected area. This change in detected intensity causes diverse resulting sums computed within presented 2D intensity signal processing of speckle patterns detected before and after displacement. Subsequently, both acquired 1D intensity signals (column vectors) can be even uncorrelated and position of maximum of normalized cross-correlation function *r*_12_ of the intensity signals does not correspond to real speckle pattern displacement; that is, *A*_*y*′_ = 0 **μ**m. Although, as the processed area of the matrix detector increases (i.e., number *n*_*s*_ of columns increases), the change in the intensity values can be within 2D intensity signal processing neglected. Subsequently, as is shown in Figures 7 and 10, both acquired 1D intensity signals become increasingly correlated with number *n*_*s*_ of columns and then the obtained values of *a*_*y*_ are equal to the theoretical one (*α*_*y*′_ = 0 **μ**m). The same principle can be generalized for any in-plane translation = (*a*_*x*_, *a*_*y*_) (see Figures 5, 6, 8, and 9).

### 3.3. Optimization of the Presented 2D Signal Processing

The previous Section 3.2 of the paper deals with evaluation of both object's translation components *a*_*x*_ and *a*_*y*_ by means of 1D cross-correlation function of intensity signals *I*_1_ and *I*_2_, which are obtained through 2D intensity signal processing of speckle pattern detected before and after object's translation. It is shown that one can use only selected part of the matrix detector to achieve approximately same results as for the whole matrix detector, as is illustrated in the graphs (Figures 5–10).

This section is focused on reduction of intensity values in detected 2D intensity signal by means of its decimation by a factor Δ. The factor Δ represents distance between rows (columns) of the detector, which are used in the 2D intensity signal processing. The 1D intensity signal acquired from 2D intensity signal processing of the decimated signal is then computed as *I*(*i*) = ∑_*j*=0_^*m*_*s*_^ *I*_(*j*Δ+1)*i*_ or *I*(*j*) = ∑_*i*=0_^*n*_*s*_^ *I*_*j*(*i*Δ+1)_, where *m* = Floor(*m*_*s*_/Δ) − 1, *n* = Floor(*n*_*s*_/Δ) − 1 and Floor (*x*) gives the greatest integer less than or equal to *x*.

**Figure 11:**
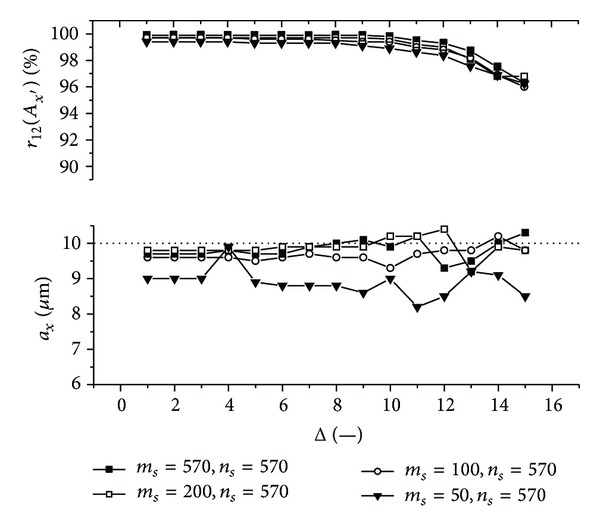
The evaluated object's translation component *a*_*x*_ as well as the maximum *r*_12_(*A*_*x*′_) of the 1D normalized cross-correlation function of intensity signals *I*_1_ and *I*_2_ acquired from decimated 2D intensity signals of various size as a function of the factor Δ. Mean speckle sizes are *α*_*x*′_ = 264.9 **μ**m and *α*_*y*′_ = 225.5 **μ**m. The object is translated by *a*_*x*_ = 10 **μ**m and *a*_*y*_ = 10 **μ**m.

**Figure 12:**
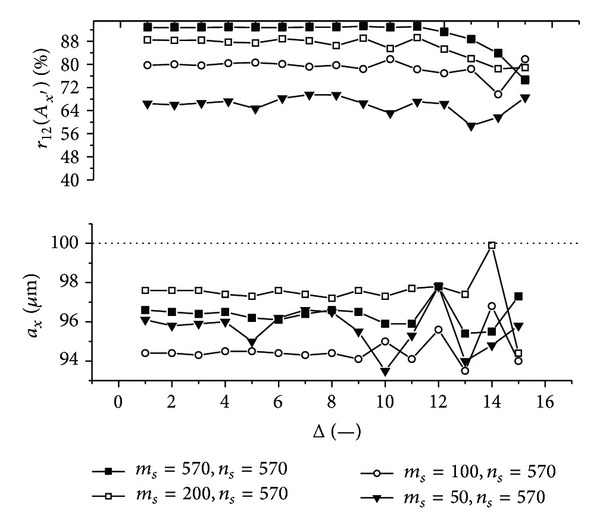
The evaluated object's translation component *a*_*x*_ as well as the maximum *r*_12_(*A*_*x*′_) of the 1D normalized cross-correlation function of intensity signals *I*_1_ and *I*_2_ acquired from decimated 2D intensity signals of various size as a function of the factor Δ. Mean speckle sizes are *α*_*x*′_ = 264.9 **μ**m and *α*_*y*′_ = 225.5 **μ**m. The object is translated by *a*_*x*_ = 100 **μ**m and *a*_*y*_ = 100 **μ**m.

**Figure 13:**
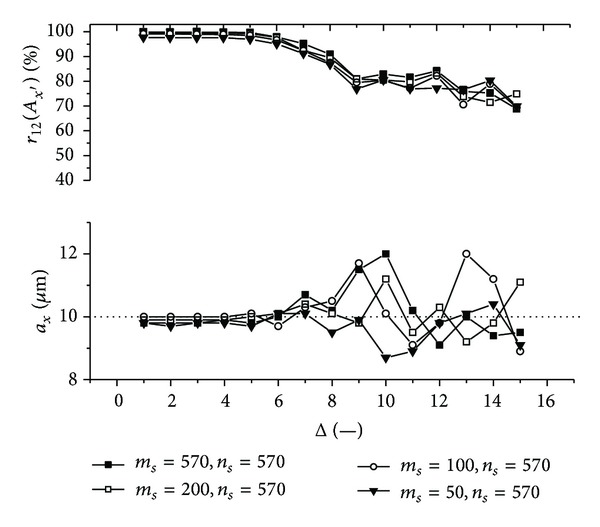
The evaluated object's translation component *a*_*x*_ as well as the maximum *r*_12_(*A*_*x*′_) of the 1D normalized cross-correlation function of intensity signals *I*_1_ and *I*_2_ acquired from decimated 2D intensity signals of various size as a function of the factor Δ. Mean speckle sizes are *α*_*x*′_ = 133.6 **μ**m and *α*_*y*′_ = 117.7 **μ**m. The object is translated by *a*_*x*_ = 10 **μ**m and *a*_*y*_ = 10 **μ**m.

**Figure 14:**
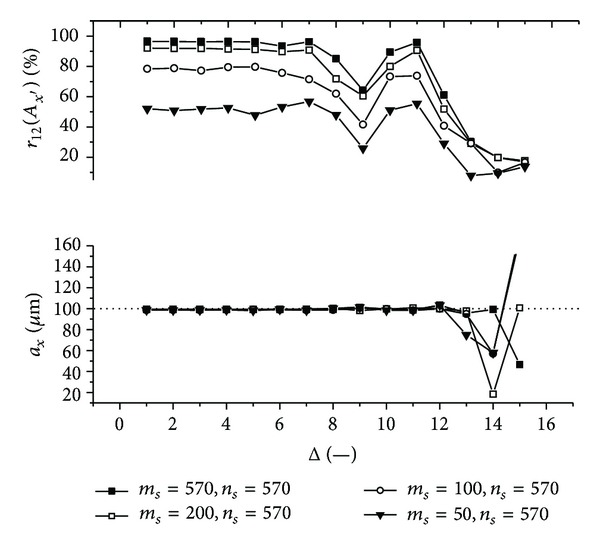
The evaluated object's translation component *a*_*x*_ as well as the maximum *r*_12_(*A*_*x*′_) of the 1D normalized cross-correlation function of intensity signals *I*_1_ and *I*_2_ acquired from decimated 2D intensity signals of various size as a function of the factor Δ. Mean speckle sizes are *α*_*x*′_ = 133.6 *μ*m and *α*_*y*′_ = 117.7 **μ**m. The object is translated by *a*_*x*_ = 100 **μ**m and *a*_*y*_ = 100 **μ**m.

**Figure 15:**
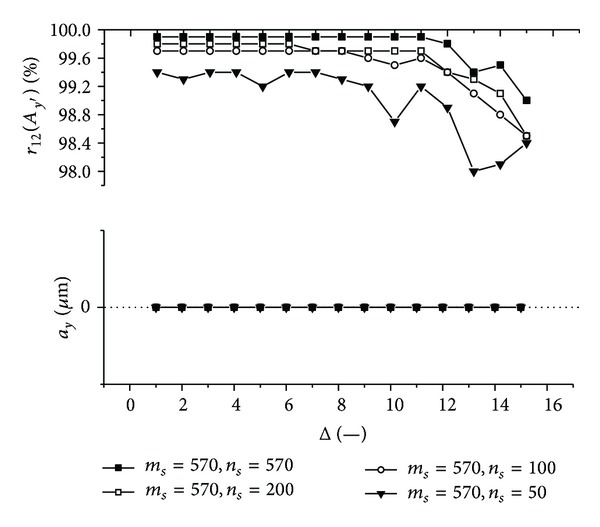
The evaluated object's translation component *a*_*y*_ as well as the maximum *r*_12_(*A*_*y*′_) of the 1D normalized cross-correlation function of intensity signals *I*_1_ and *I*_2_ acquired from decimated 2D intensity signals of various size as a function of the factor Δ. Mean speckle sizes are *α*_*x*′_ = 264.9 **μ**m and *α*_*y*′_ = 225.5 **μ**m. The object is translated by *a*_*x*_ = 10 **μ**m and *a*_*y*_ = 0 **μ**m.

**Figure 16:**
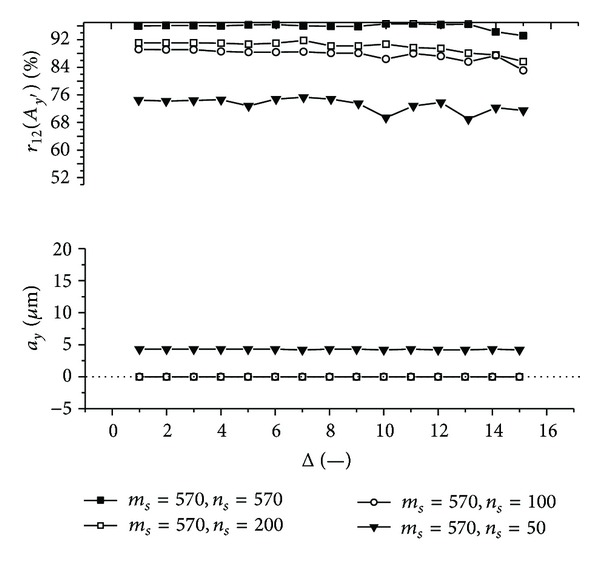
The evaluated object's translation component *a*_*y*_ as well as the maximum *r*_12_(*A*_*y*′_) of the 1D normalized cross-correlation function of intensity signals *I*_1_ and *I*_2_ acquired from decimated 2D intensity signals of various size as a function of the factor Δ. Mean speckle sizes are *α*_*x*′_ = 264.9 **μ**m and *α*_*y*′_ = 225.5 **μ**m. The object is translated by *a*_*x*_ = 100 **μ**m and *a*_*y*_ = 0 **μ**m.

**Figure 17:**
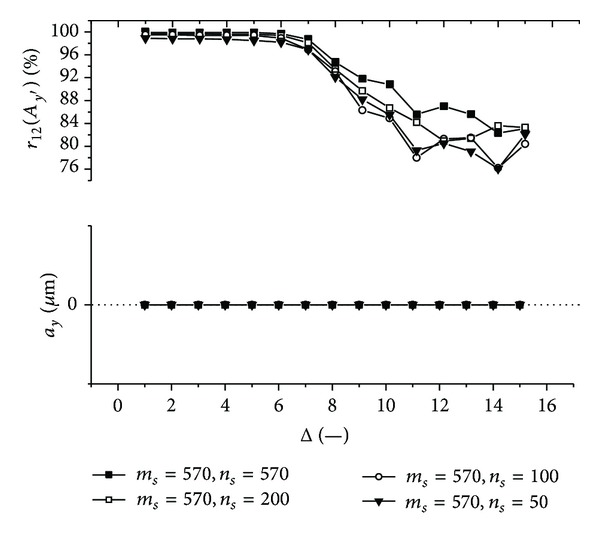
The evaluated object's translation component *a*_*y*_ as well as the maximum *r*_12_(*A*_*y*′_) of the 1D normalized cross-correlation function of intensity signals *I*_1_ and *I*_2_ acquired from decimated 2D intensity signals of various size as a function of the factor Δ. Mean speckle sizes are *α*_*x*′_ = 133.6 **μ**m and *α*_*y*′_ = 117.7 **μ**m. The object is translated by *a*_*x*_ = 10 **μ**m and *a*_*y*_ = 0 **μ**m.

**Figure 18:**
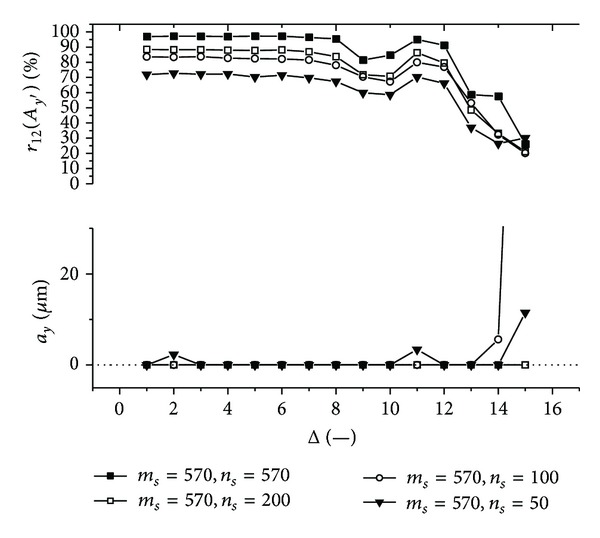
The evaluated object's translation component *a*_*y*_ as well as the maximum *r*_12_(*A*_*y*′_) of the 1D normalized cross-correlation function of intensity signals *I*_1_ and *I*_2_ acquired from decimated 2D intensity signals of various size as a function of the factor Δ. Mean speckle sizes are *α*_*x*′_ = 133.6 **μ**m and *α*_*y*′_ = 117.7 **μ**m. The object is translated by *a*_*x*_ = 100 **μ**m and *a*_*y*_ = 0 **μ**m.

Graphs (Figures 11–18) show behavior of both evaluated object's translation components *a*_*x*_ and *a*_*y*_ and the maxima of 1D normalized cross-correlation function *r*_12_ of signals *I*_1_ and *I*_2_ as a function of the factor Δ for the object translated by *a*_*x*_ = *a*_*y*_ = 10 **μ**m and *a*_*x*_ = *a*_*y*_ = 100 **μ**m (Figures 11–14) and by *a*_*x*_ = 10 **μ**m and *a*_*x*_ = 100 **μ**m, and always *a*_*y*_ = 0 **μ**m (Figures 15–18). The graphs in Figures 11, 12, 15, and 16 stand for larger speckle sizes (*α*_*x*′_ = 264.9 **μ**m and *α*_*y*′_ = 225.5 **μ**m) and the graphs in Figures 13, 14, 17, and 18 stand for smaller speckle sizes (*α*_*x*′_ = 133.6 **μ**m and *α*_*y*′_ = 117.7 **μ**m). In addition, each graph contains four curves corresponding to various size *m*_*s*_ × *n*_*s*_ of the submatrix (570 × 570, 200 × 570, 100 × 570, 50 × 570 or 570 × 570, 570 × 200, 570 × 100, 570 × 50) used for intensity signal decimation and other processing.

**Figure 19:**
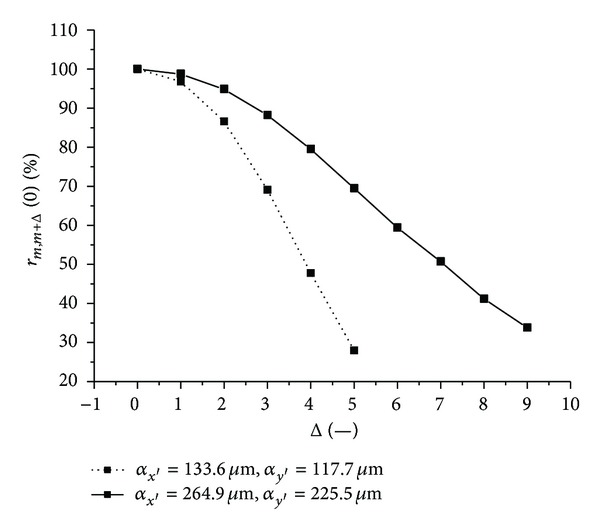
The correlation degree *r*_*m*,*m*+Δ_(0) of the 1D normalized cross-correlation function of intensity signals from the rows *m* and *m* + Δ of the matrix detector as a function of the factor Δ for both larger (*α*_*x*′_ = 264.9 **μ**m and *α*_*y*′_ = 225.5 **μ**m) and smaller (*α*_*x*′_ = 133.6 **μ**m and *α*_*y*′_ = 117.7 **μ**m) speckle sizes.

Let us discuss results obtained from the above-mentioned graphs depicted in Figures 11–18. In the case of larger speckle sizes, the maxima *r*_12_(*A*_*x*′_) and *r*_12_(*A*_*y*′_) of the normalized cross-correlation function *r*_12_ decrease slower as the factor Δ increases. This behavior can be explained as follows. For larger speckles, a 1D intensity signal is not changed so dramatically from row to row (column to column) of the matrix detector, but rather neighbouring rows (columns) are more similar to each other. This fact can be expressed via cross-correlation function of intensities of the rows (columns). For illustration, Figure 19 shows behavior of the correlation degree *r*_*m*,*m*+Δ_(0) of the 1D normalized cross-correlation function of intensity signals from the rows *m* and *m* + Δ of the matrix detector as a function of the factor Δ for both larger (*α*_*x*′_ = 264.9 **μ**m and *α*_*y*′_ = 225.5 **μ**m) and smaller (*α*_*x*′_ = 133.6 **μ**m and *α*_*y*′_ = 117.7 **μ**m) speckle sizes. One can see that the speckle pattern with larger speckles has better correlation properties. That is why the decimation process has less influence on the other successive processing of 2D intensity signal.

On the other hand, less accurate results are achieved for the case of larger speckle sizes. It is obvious, because the decimation does not improve the accuracy of evaluation of translation components, which is, as already mentioned, less for larger speckle sizes than for smaller speckle sizes (cf. e.g., Figure 11 with Figure 13). Therefore, let us further focus purely on the smaller speckles; that is, *α*_*x*′_ = 133.6 **μ**m and *α*_*y*′_ = 117.7 **μ**m (Figures 13, 14, 17, and 18).

Firstly, let us discuss an impact of the factor Δ on the achieved results. Apparently, the best results are obtained for Δ ≤ 6. Thus the decimation of the 2D intensity signal by the factor Δ = 6 still does not depreciate the results.

Further, let us compare results in Figures 13, 14, 17, and 18 obtained from various sizes *m*_*s*_ × *n*_*s*_ of the submatrix within the range Δ ∈ [1–6]. It is noted that the maxima *r*_12_(*A*_*x*′_) and *r*_12_(*A*_*y*′_) of the normalized cross-correlation function *r*_12_ of intensities *I*_1_ and *I*_2_ decrease as the size *m*_*s*_ × *n*_*s*_ decreases, although, for minimum size 570 × 50 or 50 × 570 of the submatrix, the values of the maxima *r*_12_(*A*_*x*′_) and *r*_12_(*A*_*y*′_) are still sufficiently high (the lowest values of the maximum *r*_12_(*A*_*x*′_) stand for *a*_*x*_ = 100 **μ**m and *a*_*y*_ = 100 **μ**m (Figure 14) and achieve approximately 50%).

Let us suppose that evaluation of the object's translation component *a*_*x*_ (*a*_*y*_) is reliable for *r*_12_(*A*_*x*′_) ≥ 50% (*r*_12_(*A*_*y*′_) ≥ 50%). Then the above-mentioned results imply that the minimum number of rows (columns) of the detector which one can evaluate the translation component *a*_*x*_ (*a*_*y*_) from is *m*_*s*_/Δ = 50/6≅8 (*n*_*s*_/Δ = 50/6≅8). Hence, to get reasonable results of evaluation of both translation components *a*_*x*_ and *a*_*y*_ within the interval [10–100] **μ**m for the mean speckle sizes *α*_*x*′_ = 133.6 **μ**m and *α*_*y*′_ = 117.7 **μ**m, one can select only 8 rows and 8 columns from the whole matrix detector with number of points 570 × 570. Good results are then achieved from relatively small amount of intensity values.

## 4. Conclusion

In this paper, an extension of one-dimensional speckle correlation method, which is primarily intended for determination of one-dimensional object's translation, for detection of general in-plane object's translation is presented. The numerical model for simulation of detection of translation components of the object by the speckle correlation method is used. For comparison, two speckle patterns with different mean speckle sizes are investigated. For evaluation of each of the translation components, the 1D cross-correlation function of two intensity signals *I*_1_ and *I*_2_, which are obtained by the proposed 2D intensity numerical processing of speckle patterns detected before and after object's translation, is presented. To optimize the 2D numerical processing, detected 2D speckle patterns are in addition decimated.

Some achieved results of the numerical simulation imply that the proposed 1D correlation method could successfully replace time-consuming 2D correlation method. Used decimation of intensity values enables evaluation of the translation components of the object under test from few rows and columns of the matrix detector, which can positively influence data processing speed. Moreover, since some types of matrix detectors are able to decimate the 2D signal directly from the area of the detector, then less amount of data from the detector to computer can be transferred, which can additionally improve the effectiveness of the data processing. The evaluated translation components acquired by means of the proposed method are in good agreement with the appropriate translation components computed by theoretical relations.

## Figures and Tables

**Table 1:** Object's translation components *a*_*x*_ and *a*_*y*_ computed from acquired positions *A*_*x*′_ and *A*_*y*′_ of the maxima *r*_12_(*A*_*x*′_) and *r*_12_(*A*_*y*′_) of normalized cross-correlation function *r*_12_ of intensities *I*_1_ and *I*_2_ by means of (4) and (5). Initial parameters of the computer simulation are according to Figure 1: *V*_*x*_ = *V*_*y*_ = 4 mm, *V*_*x*′_ = *V*_*y*′_ = 8 mm, *m*_*i*_ = *n*_*i*_ = 400, *m* = *n* = 570, *L*_*o*_ = 0.4 m, *L*_*s*_ = 0.2 m, *θ*_*o*_ = 30°, *ω*_*o*_ = 60 *μ*m, and *λ* = 632.8 nm. Mean speckle size of detected speckle pattern oriented along *x*′*-* and *y*′*-*axes direction is *α*_*x*′_ = 264.9 *μ*m and *α*_*y*′_ = 225.5 *μ*m. For the sake of clarity, the values of object's translation components (columns 1 and 2) and corresponding evaluated values (columns 9 and 10) are highlighted by bold font.

Object's translation		Theoretical speckle displacement		Detected speckle displacement		Maximum of cross-correlation function		Evaluated object's translation	
*a*_*x*_ (*μ*m)	*a*_*y*_ (*μ*m)	*A*_*x*′_^theor^ (*μ*m)	*A*_*y*′_^theor^ (*μ*m)	*A*_*x*′_ (*μ*m)	*A*_*y*′_ (*μ*m)	*r*_12_(*A*_*x*′_) (%)	*r*_12_(*A*_*y*′_) (%)	*a*_*x*_ (*μ*m)	*a*_*y*_ (*μ*m)
**10**	**0**	31.8	0.0	31.5	0.0	99.9	96.4	**9.9**	**0.0**
**20**	**0**	63.5	0.0	62.9	0.0	99.9	87.9	**19.8**	**0.0**
**30**	**0**	95.3	0.0	94.4	0.0	99.9	74.1	**29.7**	**0.0**
**40**	**0**	127.0	0.0	125.9	10.8	99.7	55.2	**39.6**	**3.6**
**50**	**0**	158.8	0.0	157.4	14.6	99.6	31.3	**49.6**	**4.9**
**60**	**0**	190.5	0.0	188.8	12.8	99.4	9.1	**59.4**	**4.3**
**70**	**0**	222.3	0.0	220.3	436.8	99.2	6.6	**69.4**	**145.6**
**80**	**0**	254.0	0.0	251.8	451.1	99.0	11.8	**80.0**	**150.4**
**90**	**0**	285.8	0.0	283.3	473.2	98.7	17.4	**90.0**	**157.7**
**100**	**0**	317.5	0.0	314.8	497.1	98.4	23.2	**100.0**	**165.7**
**10**	**10**	31.8	30.0	33.1	31.2	94.8	93.4	**10.4**	**10.4**
**20**	**20**	63.5	60.0	70.2	62.7	75.1	88.0	**22.1**	**20.9**
**30**	**30**	95.3	90.0	112.4	94.6	49.6	73.9	**35.4**	**31.5**
**40**	**40**	127.0	120.0	162.4	126.3	31.4	54.4	**51.1**	**42.1**
**50**	**50**	158.8	150.0	255.9	156.2	23.4	30.6	**80.6**	**52.1**
**60**	**60**	190.5	180.0	417.0	176.0	31.2	9.4	**131.3**	**58.7**
**70**	**70**	222.3	210.0	460.5	112.3	37.4	0.2	**145.0**	**37.4**
**80**	**80**	254.0	240.0	494.7	112.3	36.0	1.6	**155.8**	**37.4**
**90**	**90**	285.8	270.0	525.6	561.4	30.7	4.2	**165.5**	**187.1**
**100**	**100**	317.5	300.0	561.4	561.4	20.3	4.1	**176.8**	**187.1**

**Table 2:** Object's translation components *a*_*x*_ and *a*_*y*_ computed from acquired positions *A*_*x*′_ and *A*_*y*′_ of the maxima *r*_12_(*A*_*x*′_) and *r*_12_(*A*_*y*′_) of normalized cross-correlation function *r*_12_ of intensities *I*_1_ and *I*_2_ by means of (4) and (5). Initial parameters of the computer simulation are according to Figure 1: *V*_*x*_ = *V*_*y*_ = 4 mm, *V*_*x*′_ = *V*_*y*′_ = 8 mm, *m*_*i*_ = *n*_*i*_ = 400, *m* = *n* = 570, *L*_*o*_ = 0.4 m, *L*_*s*_ = 0.2 m, *θ*_*o*_ = 30°, *ω*_*o*_ = 60 *μ*m, and *λ* = 632.8 nm. Mean speckle size of detected speckle pattern oriented along *x*′*-* and *y*′*-*axes direction is *α*_*x*′_ = 133.6 *μ*m and *α*_*y*′_ = 117.7 *μ*m. For the sake of clarity, the values of object's translation components (columns 1 and 2) and corresponding evaluated values (columns 9 and 10) are highlighted by bold font.

Object's translation		Theoretical speckle displacement		Detected speckle displacement		Maximum of cross-correlation function		Evaluated object's translation	
*a*_*x*_ (*μ*m)	*a*_*y*_ (*μ*m)	*A*_*x*′_^theor^ (*μ*m)	*A*_*y*′_^theor^ (*μ*m)	*A*_*x*′_ (*μ*m)	*A*_*y*′_ (*μ*m)	*r*_12_(*A*_*x*′_) (%)	*r*_12_(*A*_*y*′_) (%)	*a*_*x*_ (*μ*m)	*a*_*y*_ (*μ*m)
**10**	**0**	31.8	0.0	31.7	0.0	99.9	82.9	**10.0**	**0.0**
**20**	**0**	63.5	0.0	63.4	0.0	99.9	35.3	**20.0**	**0.0**
**30**	**0**	95.3	0.0	95.1	315.2	99.8	18.7	**30.0**	**105.1**
**40**	**0**	127.0	0.0	126.8	137.7	99.7	8.9	**39.9**	**45.9**
**50**	**0**	158.8	0.0	158.6	124.7	99.6	11.8	**49.9**	**41.6**
**60**	**0**	190.5	0.0	190.3	113.3	99.5	12.4	**59.9**	**37.8**
**70**	**0**	222.3	0.0	222.1	97.5	99.4	8.2	**70.0**	**32.5**
**80**	**0**	254.0	0.0	253.9	0.0	99.2	7.5	**80.0**	**0.0**
**90**	**0**	285.8	0.0	285.6	0.0	99.1	12.7	**90.0**	**0.0**
**100**	**0**	317.5	0.0	317.3	0.0	98.9	16.0	**99.9**	**0.0**
**10**	**10**	31.8	30.0	28.5	30.1	63.8	81.9	**9.0**	**10.0**
**20**	**20**	63.5	60.0	54.5	57.4	12.6	37.1	**17.2**	**19.1**
**30**	**30**	95.3	90.0	321.5	407.6	6.8	17.8	**101.2**	**135.9**
**40**	**40**	127.0	120.0	343.6	249.8	21.3	10.7	**108.2**	**83.3**
**50**	**50**	158.8	150.0	381.6	265.3	29.4	13.5	**120.2**	**88.4**
**60**	**60**	190.5	180.0	417.3	274.0	27.3	15.9	**131.4**	**91.3**
**70**	**70**	222.3	210.0	0.0	216.9	19.2	14.1	**0.0**	**72.3**
**80**	**80**	254.0	240.0	441.8	193.0	9.8	18.7	**139.1**	**64.6**
**90**	**90**	285.8	270.0	318.5	222.2	7.1	23.6	**100.3**	**73.7**
**100**	**100**	317.5	300.0	505.3	261.5	3.4	19.7	**159.1**	**87.2**
